# Characterization of the effect of *cis*-3-hexen-1-ol on green tea aroma

**DOI:** 10.1038/s41598-020-72495-5

**Published:** 2020-09-23

**Authors:** Cong-ning Nie, Yuan Gao, Xiao Du, Jin-lin Bian, Hui Li, Xiang Zhang, Cong-ming Wang, Shun-yu Li

**Affiliations:** Sichuan Agricultural University, No. 211 Huimin Road, Wenjiang District, Chengdu, 610000 Sichuan China

**Keywords:** Biochemistry, Plant sciences

## Abstract

*cis*-3-Hexen-1-ol has been regarded as the main source of green aroma (or green odor) in green tea. However, no clear findings on the composition of green aroma components in tea and the effect of *cis*-3-hexen-1-ol on other aroma components have been reported. In this study, the main green aroma components in green tea were characterized, especially the role of *cis*-3-hexen-1-ol in green aroma was analyzed and how it affected other aroma components in green tea was studied. Based on the GC–MS detection, odor activity value evaluation, and monomer sniffing, 12 green components were identified. Through the chemometric analysis, *cis*-3-hexen-1-ol was proven as the most influential component of green aroma. Moreover, through the electronic nose analysis of different concentrations of *cis*-3-hexen-1-ol with 25 other aroma components in green tea, we showed that the effect of *cis*-3-hexen-1-ol plays a profound effect on the overall aroma based on the experiments of reconstitution solution and natural tea samples. GC–MS and CG-FID confirmed that the concentration range of the differential threshold of green odor and green aroma of *cis*-3-hexen-1-ol was 0.04–0.52 mg kg^−1^.

## Introduction

*cis*-3-Hexen-1-ol is one of the most important VOCs in plants^1^. It was widely found in fresh leaves of tea as an important allelochemicals responding to mechanical injury^2,3^. Many studies have been carried out to reveal its allelopathic effects on plants, pests, and natural enemies from previous reports^4,5^. *cis*-3-Hexen-1-ol is also a significantly important active-component which contributes to the “green, grass and fresh” odor in vegetable food, such as grapes^6^, passion fruits^7^, and Toona sinensis^8^. Though tea is one of the largest consumption drink in human being’s life, no previous studies have been devoted to study the contribution of *cis*-3-hexen-1-ol on the favorite aroma of tea. On the other hand, *cis*-3-hexen-1-ol could also plays a determining role as the “raw grass” odor of finished green tea due to its over strong and sharp green aroma. Therefore, these observations highlight the importance to systematically study the effects of *cis*-3-hexen-1-ol on the overall aroma of finished tea products. Due to the strong feedback of *cis*-3-hexen-1-ol in the odor system, it could be also important to study the effect of *cis*-3-hexen-1-ol in the mixture with other odor components in the green tea.

As mentioned before, “raw grass” odor is thought to have a negative effect on the aroma of green tea, thus *cis*-3-hexen-1-ol of green tea should be controlled in a very low range (descriptive evaluation considers that it should be as low as unrecognizable). In manufacturing process, most of *cis*-3-hexen-1-ol is volatilized and/or converted into other aroma components (e.g. *trans*-3-hexen-1-ol, jasmone^9^) due to high temperature in sterilization and drying. The volatilization of low-boiling aroma components, like 1-pentanol (fruity), citronellol (floral) and linalool (floral), weakens silky and refreshing flavor of tea^10^. Meanwhile, Maillard reaction^11^ might occur in manufacturing process, in which the overall aroma of tea will be altered into roast and chestnut-like aroma. Whether *cis*-3-hexen-1-ol plays the most important role in providing green odor of tea and how it affects the tea aroma are still unclear and further investigations are needed.

In recent years, some studies about the influences of *cis*-3-hexen-1-ol on the aroma have been reported^12,13^. However, most of the researches were devoted to detection, qualitative and quantitative analysis, and rare studies focused on the effects of interactions of different components on the tea odor^14^. To our best knowledge, no study about the components of green aroma of tea and how *cis*-3-hexen-1-ol affects the overall aroma of tea have been reported. Further investigations are needed to clarify that whether *cis*-3-hexen-1-ol at different concentrations synergize or mask other aroma components in the mixed system of finished tea, and how it fluctuates from raw green odor (high concentration) to green aroma (low concentration).

In this study, the identification of green aroma (odor)- producing components and dominant components of green tea were studied by four steps: (1) qualitative and quantitative analysis of VOCs of green tea in different processing stages by HS-SPME-GC–MS/GC-FID; (2) evaluation about the conversion process of green tea from green odor to green aroma by sensory evaluation and E-nose; (3) evaluation, screening and ranking of the aroma active components of finished tea by the OAV and aroma monomer sniffing; (4) correlation analysis and stepwise linear regression. In addition, effect pattern and ability of *cis*-3-hexen-1-ol on the green tea aroma was studied in 3 steps.: (1) validation of the effectiveness of aroma active components by reconstitution experiment; (2) evaluation of the effects of different concentrations of *cis*-3-hexen-1-ol on other aroma components in monomer system by mixing different concentrations of *cis*-3-hexen-1-ol with each aroma component respectively; (3) evaluation of the effects of *cis*-3-hexen-1-ol on aroma profile in tea system by addition of gradient concentration of *cis*-3-hexen-1-ol to finished tea without green odor. Herein, the effects of *cis*-3-hexen-1-ol on the aroma of green tea were characterized, and a new strategy for studying the effects of specific aroma component on the whole aroma profile in food was presented.

## Method and material

### Materials

#### Tea samples and process parameters

The raw materials (fresh tea buds) were taken from 5-year-old tea trees (*Camellia sinensis*) which cultivar was Fuxuan No. 9, the standard for picking was whole bud from the same batch, and tea garden located in Mingshan District (N30° 17′, E103° 21′).

Above raw materials were processed immediately in the production line of "Green Bud Tea" in a tea factory in Mingshan District, and the samples were taken at five processing stages: spreading (sample A), fixing (sample B), carding (sample C), pressing flat (sample D) and drying (finished tea) (sample E). Sampling 250 g at each processing stage was put into a refrigerator at – 40 °C for freezing storage (in March 2018).

Specific process parameters were as follows:

Spreading (light withering), which step that put tea buds in withering trough and withered lightly for 10 h (the thickness was 8 cm and the temperature was 17 ± 2 °C).

Fixing, which step that inactivated enzyme activity of tea buds using infrared plate-type fixing machine, the set temperature was 105 °C (10 min), and measured temperature was 69.30 ± 0.39 °C. After fixing, tea buds were cooled for 10 min to 20 ± 2 °C.

Carding, which step that the tea buds were stir-fried at low temperature to volatilize water and developed aroma while straightening out the shape by a carding machine (Supplementary Annex S1), the set temperature was 100 °C (45 min), frequency was 107 r min^−1^, and measured temperature was 46.27 ± 0.28 °C.

**Figure 1:**
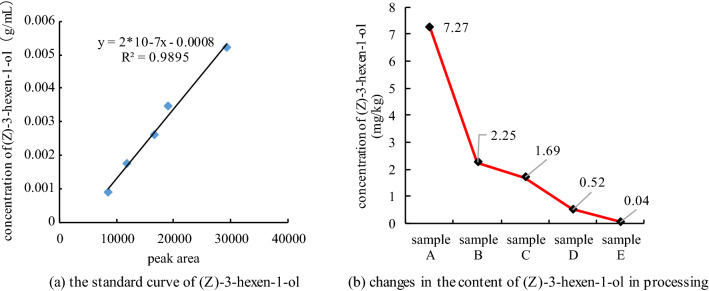
Standard curve of (Z)-3-hexen-1-ol and its content change in processing.

Pressing flat, which step that added pressure bars (the length, diameter and mass of each bar were 60 cm, 3.2 cm and 1.1 kg respectively) (Fig. 1) in the above carding machine to flat tea buds, the set temperature was 68 °C (10 min), frequency was 50 r min^−1^, and measured temperature was 26.30 ± 0.22 °C.

Drying, which step that the tea buds were dried at low temperature for a long time with the above carding machine, and the finished tea (water content less than 5%, m/m) was finally obtained, the set temperature was 66 °C (260 min), frequency was 80 r min^−1^, and measured temperature was 40.90 ± 0.67 °C.

#### Chemicals and reagents

*cis*-3-Hexen-1-ol and ether standard (purity > 99.97%), which were purchased from Sigma-Aldrich (Shanghai, China), were used for GC-FID quantitative analysis, and GC–MS qualitative analysis with recovery ratio correction; C4–C20 normal paraffin standards (purity > 99.97%, used for RI calculation) was purchased from Sigma-Aldrich (Shanghai, China); hexanal, myrcene, 1-penten-3-ol, 2-hexenal (mixture of *cis* and trans isomers, molar ratio about 1:1), 1-pentanol, trans-2-heptenal, 1-hexanol, (E)-3-hexen-1-ol, *cis*-3-hexen-1-ol, 1-octen-3-ol, furanoid-linalool oxide (mixture of *cis* and trans isomers, molar ratio about 1:1), pyran-linalool oxide (mixture of *cis* and trans isomers, molar ratio about 1:1), benzaldehyde, 1-ethyl-1H-pyrrole-2-carboxaldehyde, linalool, phenylacetaldehyde, citral, dodecanal, methyl salicylate, citronellol, nerol, geraniol, jasmone, 1-tetradecylaldehyde, above 24 aroma components (purity > 98%) which were purchased from Rhawn reagent (Shanghai, China), were used for aroma profile test and reconstitution experiment.

#### Equipment

HS-SPME was used to extract the aroma compounds. The SPME fibre was 50/30 μm DVB/CAR/PDMS which length was 1 cm (Sigma-Aldrich, Shanghai, China), and the fiber penetration depth into the headspace was 2 cm. The size of the extraction bottle was 15 mL.

GC-FID was used for quantitative analysis, which was a Shimadzu GC-2014 (Kyoto, Japan) fitted with FID-2014 flame ionization detector.

GC–MS was used for qualitative analysis, which was a Shimadzu QP2010 SE (Kyoto, Japan).

E-nose was used to assist sensory evaluation, which was a FOX 4000 from Alpha-MOS (Toulouse, France) including 18 different metal oxide semiconductor gas sensors, combined with a headspace auto-sampler.

### Methods

#### Measurements conditions of HS-SPME and GC–MS

##### HS-SPME conditions and extracting method

The SPME fibre was 50/30 μm DVB/CAR/PDMS which length was 1 cm (Sigma-Aldrich, Shanghai, China), and the fiber penetration depth into the headspace was 2 cm. The size of the extraction bottle was 15 mL.

When extracting aroma substances, a tea sample (5.0 g) was placed in an extraction bottle, which was equilibrated in a thermostatic water bath for 10 min at 80 °C and then sampled for 30 min in the head space. After that, SPME fiber was then withdrawn and directly introduced to the GC–MS, and the process was repeated 3 times.

##### GC–MS conditions

Capillary–column chromatography was DB-WAX (30 m × 250 μm × 0.25 μm) (Agilent), the sampling was manual, no shunt was used, the sample was at a constant temperature, and the temperature of the injection port and GC–MS direct interface were both 250 °C, helium was used as a carrier gas at a flow rate of 1.67 mL min^−1^. Temperature programming: the column temperature was 45–220 °C; the starting column temperature was 45 °C, which was held for 3 min, increased to 180 °C at a rate of 3 °C min^−1^, held for 3 min, then increased to 220 °C at a rate of 10 °C min^−1^, and held for 3 min.

The ionic source temperature was 220 °C, its electron ionization energy was 70 eV, the quadrupole mass filter was operated at 150 °C, and the scanning quality range was set at 20–700 amu.

##### Qualitative analysis of aroma compounds

Qualitative analysis: the NIST Standard spectral library (https://www.nist.gov/) and retention indices (RIs) from other literature were used to match ion mass spectra, then completed qualitative analysis of aroma components:

1 $$ {\text{The RI calculation formula was}}\;RI = 100{\text{n}} + 100 \times \frac{RTx - RTn}{{RTn + 1 - RTn}} $$

where RI, were retention indices of analyte, n, the number of carbon atoms of n-alkanes before the analyte, RT_x_, the retention time of the analyte, RT_n_, the retention time of n-alkanes before the analyte, RT_n+1_, the retention time of n-alkanes after the analyte.

#### Measurements conditions of GC-FID

##### HS-SPME conditions and extracting method

Same as “HS-SPME conditions and extracting method”.

##### GC-FID conditions

Capillary-column chromatography was AT-SE-54 (30 m × 250 μm × 0.33 μm), and the detector was hydrogen flame ionization detector (FID). The sampling was manual, no shunt was used, the sample was at a constant temperature, and the temperature of the injection port and the detector were both 230 °C, nitrogen was used as a carrier gas at a flow rate of 1.00 mL min^−1^. Temperature programming: the column temperature was 40–230 °C; the starting column temperature was 40 °C, which was held for 2 min, increased to 110 °C at a rate of 2 °C min^−1^, held for 2 min, then increased to 130 °C at a rate of 7 °C min^−1^, and held for 2 min, finally, increased to 230 °C at a rate of 5 °C min^−1^, and held for 8 min.

##### Standard curve experiment of *cis*-3-hexen-1-ol

Using ether as solvent, the gradient concentration standard solutions of *cis*-3-hexen-1-ol (V/V%) were 0.1% (1.692 mg mL^−1^), 0.2% (3.384 mg mL^−1^), 0.3% (8.46 mg mL^−1^), 0.4% (13.536 mg mL^−1^) and 0.5% (16.92 mg mL^−1^), respectively. The above standard solution was detected by GC-FID according to “HS-SPME conditions and extracting method” and “GC-FID conditions” method, then the standard curve of *cis*-3-hexen-1-ol was obtained. The experiment was repeated 3 times.

##### Qualitative and quantitative determination of *cis*-3-hexen-1-ol in tea samples.

Tea samples were obtained according to“Tea samples and process parameters”, and the peak area of *cis*-3-hexen-1-ol in tea samples was determined by “HS-SPME conditions and extracting method” and “GC-FID conditions”, qualitative determination of chlorophyll in tea samples is determined by peak time of standard samples, the content of *cis*-3-hexen-1-ol was calculated according to the standard curve of “Standard curve experiment of *cis*-3-hexen-1-ol”. The experiment was repeated 3 times.

##### Quantitative determination of other aroma components in tea samples

The content of other aroma compounds was calculated by peak area ratio of GC–MS, based on *cis*-3-hexen-1-ol content measured by 2.2.2.4 method^15^.

2 $$ {\text{The calculation formula was}}\;Wi = Ws \cdot \frac{Ai}{{As}} $$

Which *W*_*i*_, was the analyte concentration in mg kg^−1^, *W*_s_, the concentration of measured *cis*-3-hexen-1-ol content in mg kg^−1^, *A*_*i*_, the analyte area, and *A*_*s*_, the peak area of *cis*-3-hexen-1-ol.

#### Measurements conditions of E-nose

The detection method of E-nose was based on the method of Qin^16^, and the visualization analysis method of E-nose was based on the method of Zhu^17^. Detector information of the E-nose was detailed in Supplementary Annex S2.

#### Effects of different concentrations of *cis*-3-hexen-1-ol on the strength of aroma monomers in tea samples

Based on the types and contents of aroma compounds detected in finished tea (sample E) which was detected from GC–MS, the double concentrations of each monomer in aqueous solution (mg kg^−1^) were prepared respectively (except *cis*-3-hexen-1-ol). Then the concentration of *cis*-3-hexen-1-o in sample E was treated as 100%, then 20%, 100%, 200%, 400%, 1,000% and 2000% *cis*-3-hexen-1-ol aqueous solutions (mg kg^−1^) were prepared respectively.

Next, different concentrations of *cis*-3-hexen-1-ol solution were mixed with aroma monomer solution at 1:1 (10 mL/10 mL), thus different concentrations of *cis*-3-hexen-1-ol (10%, 50%, 100%, 200%, 500% and 1,000%) mixed with other aroma monomers were obtained.

Finally, the above solution was sniffed and graded (7-point scoring method) by a six-person (3 male with 3 female) evaluation team which trained in systematic sniffing (specific training methods refer to the previous reports of our group^18^, including two parts, strength training of aroma monomer and recognition training of mixed solution). Using distilled water (0%) as CK, room temperature 23 ± 1 °C, all experiments were repeated 3 times. The influence of *cis*-3-hexen-1-ol on aroma monomers could be judged by the strength change of aroma monomers.

#### Preparation of the reconstitution solution

The aroma reconstitution solution was prepared according to the aroma components and content of OAV > 1 detected in the finished tea sample (sample E), and the substrate was distilled water.

#### Effects of different concentrations of *cis*-3-hexen-1-ol on the overall aroma profile of finished tea and reconstitution solution

The concentration of *cis*-3-hexen-1-o in sample E was treated as 100%, each 5 g finished tea (sample E) was treated as a treatment group, and added 0% (CK, the total *cis*-3-hexen-1-o in the sample was 100%.), 10% (total was 110%), 50% (total was 150%) , 100% (total was 200%), 200% (total was 300%), 500% (total was 600%) and 1,000% (total was 1,100%) *cis*-3-hexen-1-ol respectively, in addition, the same treatment was carried out on the reconstitution solution to verify the consistency of the change of the overall aroma caused by *cis*-3-hexen-1-ol in natural tea and reconstituted solution.

Then aroma profile analysis was carried out according to the method established by our group. The chosen descriptors were floral (reference aroma monomer was 3,7-dimethyl-1,6-octadien-3-ol), fruity ((Z)-3,7-dimethyl-2,6-octadien-1-ol), fresh ((Z)-2-hexenal), green (*cis*-3-hexen-1-ol), roasted (furfural), herbal ((Z)-3-methyl-2-(2-pentenyl)-2-cyclopenten-1-one), respectively. 7-point scoring method was selected for evaluation, which were 0 (odorless), 0.5, 1, 1.5, 2, 2.5 and 3 (very strong).

#### Data processing method

Excel 2016 were used to analyse the data and draw figures, and the SPSS 25 was used to correlation analysis and stepwise linear regression, the Photoshop CS6 were used to draw the tea processing equipment, and chemDraw 18 were used to draw chemical structures.

### Ethics requirements

In the sensory evaluation part of this study, trained evaluators were used to conduct necessary sensory evaluation on tea samples. Except for the above parts, this study does not include other work with human or animal. The study was approved by the local ethics committee in China, and all methods were carried out in accordance with relevant guidelines and regulations, informed consent was obtained from all subjects before their participation in the study.

## Results and analysis

### Detection result of GC-FID

Firstly, the content of *cis*-3-hexen-1-ol in 5 tea samples was determined by GC-FID standard curve. The standard curve was shown as Fig. 1a, R2 = 0.9895, it is considered that the results of linear fitting were well, then based on the above standard curve and the results of GC-FID detection of tea samples, the content of *cis*-3-hexen-1-o in tea samples can be calculated.

**Figure 2:**
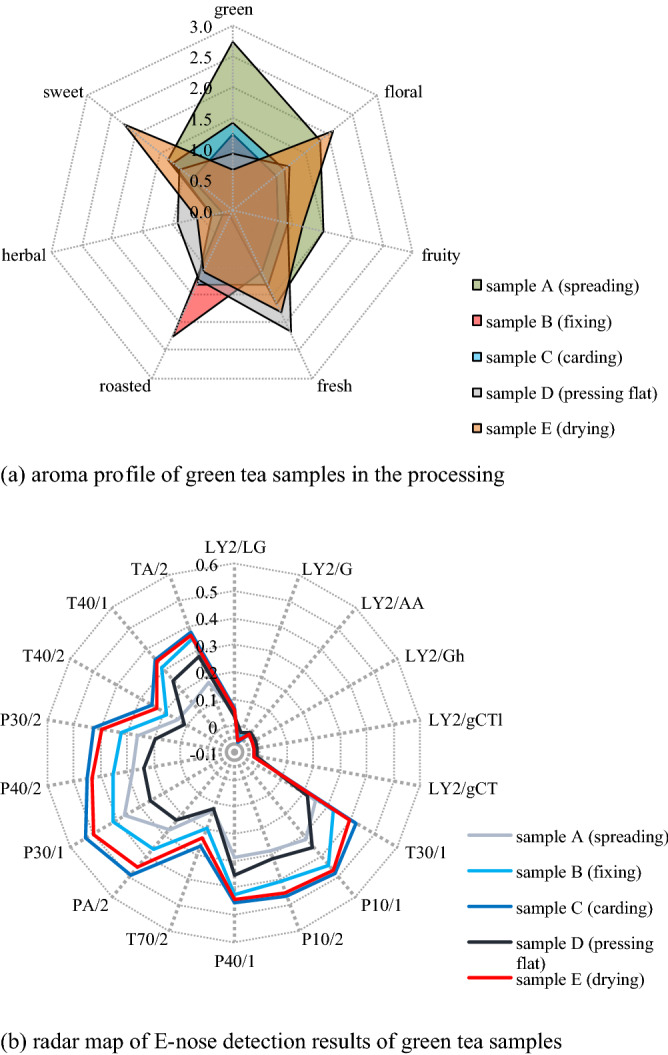
Aroma profile and E-nose radar map of green tea samples in processing.

From Fig. 1b, the *cis*-3-hexen-1-ol content of sample A was the highest (7.27 mg kg^−1^), and then decreased with the processing until sample E reached the lowest (0.04 mg kg^−1^). This result was consistent with previous studies, that is, the content of *cis*-3-hexen-1-ol formed by lipoxygenase pathway in fresh leaves will continue to decline during the processing of green tea. And there were at least 3 reasons for the decrease, first, it will gradually volatilize with the heating in the processing; and then, it will isomerize at high temperature and convert to *trans*-3-hexen-1-ol; in addition, it may be cyclized and converted into jasmone.

Thus, the concentration of *cis*-3-hexen-1-ol in different tea samples was obtained. Combined with the subsequent GC–MS detection data, the relative concentration of each aroma component in 5 tea samples could be calculated.

### Detection results of GC–MS and calculation results of OAV

The aroma compounds of different tea samples were determined by HS-SPME–GC–MS, and qualitative analysis was carried out by NIST Standard spectral library (https://www.nist.gov/) and Retention Indices (RIs), this was shown in Supplementary Annex S3 (relative peak area percentage was calculated by peak area normalization method). Then the other aroma compounds of tea samples were quantified by the content of *cis*-3-hexen-1-o determined above, meanwhile, the aroma characteristics of monomers were determined by FEMA GRAS Database^19^ (https://www.femaflavour.org/) and sensory experiments, OAV was calculated by odor threshold (OT) reviewed in Leffingwell & Associates Threshold Value Database (https://www.leffingwell.com/) and other literature. The results were shown in Table 1.

**Table 1:** Content of aroma components, OAV and aroma description of green tea samples detected and calculated by GC–MS.

No.^a^	aroma components	Concn (mg kg^−1^)^b^					OT (ppm)^c^	OAV^g^					Aroma discription^d^		
		A	B	C	D	E		A	B	C	D	E	Reference	Sniffing tests	COG^e^
33	(Z)-3-Methyl-2-(2-pentenyl)-2-cyclopenten-1-one	0.17	0.13	0.11	0.19	1.55	0.0003	559.03	424.14	352.73	625.14	5,000.00	Floral	Herb, aging, flower	0
20	3,7-Dimethyl-1,6-octadien-3-ol	6.91	3.51	2.31	1.03	2.15	0.0015	4,604.85	2,342.07	1539.57	687.65	1,435.56	Coriander, floral, lavender, lemon, rose	Floral	0
24	Dodecanal	–	0.43	0.54	0.54	0.73	0.0011	0.00	400.26	509.03	501.92	679.13	Citrus, fat, lily	Fat, oil, gardenia	0
16	Linalool oxide (pyranoid) (III and IV)	2.13	1.80	2.79	0.92	1.88	0.0060	354.92	299.48	465.81	153.44	313.33	Flower^f1^	Flower, sweet, woody	0
15	1-Octen-3-ol	0.19	0.45	0.84	1.21	0.58	0.0020	95.56	223.45	420.17	605.25	290.00	Cucumber, earth, fat, floral, mushroom	Earth, mushroom	0
7	1-Pentanol	0.09	2.97	2.84	3.06	1.32	0.0050	18.72	593.38	568.94	611.21	264.00	Fruity	Fruity, sweet	0
17	Linalool oxide (furanoid) (I and II)	–	–	0.46	0.58	1.35	0.0060	0.00	0.00	76.77	96.61	225.56	Leafy, earthy, sweet, floral, creamy^f2^	Flower, sweet, enzymatic fermentation	0
28	Geraniol	5.83	0.90	0.75	0.96	1.44	0.0075	777.96	119.59	100.01	127.87	192.00	Geranium, lemon peel, passion fruit, peach, rose	Sweet, fruit, flower, lemon, green	1
8	(E)-2-Heptenal	0.20	1.16	1.00	1.61	1.92	0.0130	15.00	89.05	76.85	123.61	147.69	Almond, fat, fruit	Almond, fat, fruit	0
1	Hexanal	0.49	0.30	0.22	0.33	0.58	0.0050	98.29	60.83	44.82	66.49	116.00	Apple, fat, fresh, green, oil	Apple, fresh, green	1
23	3,7-Dimethyl-(Z)-2,6-Octadienal	1.03	0.41	0.39	1.00	1.87	0.0300	34.19	13.55	13.07	33.25	62.44	Lemon	Lemon, sweet, fresh	0
5	(Z)-2-Hexenal	3.05	3.16	0.43	1.53	2.36	0.0400	76.35	78.98	10.66	38.15	59.00	Fresh fruit, green	Fresh fruit, green, sweet	1
4	(E)-2-Hexenal	1.84	1.78	1.64	1.39	2.31	0.0400	46.12	44.38	41.08	34.63	57.83	Fresh fruit, green	Fresh fruit, green, sweet	1
22	Benzeneacetaldehyde	–	–	–	–	0.50	0.0090	0.00	0.00	0.00	0.00	55.56	Berry, geranium, honey, nut, pungent	Honey,sweet, fermented fruit	0
25	Methyl salicylate	1.45	1.09	0.77	0.84	3.13	0.0600	24.25	18.21	12.86	13.99	52.22	Almond, caramel, peppermint, sharp	Holly oil, mint, sweet, sharp	0
2	β-Myrcene	0.55	0.09	0.42	0.43	0.67	0.0140	39.00	6.21	30.23	30.75	47.62	Balsamic, fruit, geranium, herb, must	Balsamic, fruit, herb, must	0
11	(E)-3-Hexen-1-ol	0.29	3.13	3.94	3.30	2.74	0.0700	4.12	44.78	56.29	47.13	39.14	Green	Green	1
21	1-Ethyl-1H-pyrrole-2-carboxaldehyde	–	–	–	–	1.09	0.0370	0.00	0.00	0.00	0.00	29.37	Savory	Sweet, flower, fresh tea	0
26	Citronellol	–	0.07	–	0.32	1.03	0.0400	0.00	1.86	0.00	8.10	25.67	Citrus, green, rose	Green, fresh, flower, creamy	1
19	Benzaldehyde	0.12	0.24	0.78	1.68	4.25	0.3000	0.42	0.81	2.61	5.60	14.18	Bitter almond, burnt sugar, cherry, malt, roasted pepper	Bitter almond, cherry	0
35	Tetradecanal	–	–	–	0.97	0.53	0.1100	0.00	0.00	0.00	8.80	4.85	oil, peach, iris	Peach, oil, flower	0
10	1-Hexanol	2.73	0.25	0.08	0.51	0.91	0.2500	10.92	1.01	0.31	2.05	3.63	Banana, flower, grass, herb	Green, flower, unripe banana	1
27	(Z)-3,7-Dimethyl-2,6-octadien-1-ol	0.18	0.39	0.02	0.20	0.62	0.4000	0.45	0.98	0.04	0.51	1.55	Floral, fruit	Sweet, fruit, flower, lemon	0
37	1-Hexadecanol	–	–	–	0.26	1.15	1.1000	0.00	0.00	0.00	0.23	1.04	Flower, wax	Wax, oil	0
3	1-Penten-3-ol	0.15	0.08	1.10	2.30	3.05	3.0000	0.05	0.03	0.37	0.77	1.02	Butter, fish, green, oxidized, wet earth	Wet earth, fishlike, green	1
32	Phenylethyl alcohol	0.98	–	–	–	3.81	7.5000	0.13	0.00	0.00	0.00	0.51	Fruit, honey, lilac, rose, wine	Alcohol, fruit	0
14	Furfural	0.16	0.39	0.96	1.30	2.13	8.0000	0.02	0.05	0.12	0.16	0.27	Almond, baked potatoes, bread, burnt, spice	Baked bread, bunrt	0
12	*cis*-3-Hexen-1-ol	7.27	2.25	1.69	0.52	0.04	0.2000	36.35	11.25	8.45	2.60	0.20	Grass, green fruit, green leaf, herb, unripe banana	Grass, green	1
18	2-Ethyl-1-hexanol	0.15	0.29	0.36	0.57	1.79	270.0000	0.00	0.00	0.00	0.00	0.01	Green, rose	Green, rose	1
6	3-Methyl-1-butanol	0.14	0.03	–	–	–	0.2500	0.56	0.12	0.00	0.00	0.00	Burnt, cocoa, floral, malt	Burnt, cocoa, malt	0
9	(Z)-2-Penten-1-ol	0.17	0.66	0.46	–	–	0.7200	0.23	0.92	0.64	0.00	0.00	Banana, green, rubber	Banana, green	1
13	(E)-2-Hexen-1-ol	1.42	0.80	0.43	–	–	0.6000	2.37	1.33	0.72	0.00	0.00	Blue cheese, vegetable	Fresh fruit	1
38	1-Nonadecanol	–	–	–	2.45	0.48	Null	Null	Null	Null	Null	Null	Odorless	Odorless	0
29	Propanoic acid, 2-methyl-3-hydroxy-2,4,4-trimethyl-pentyl ester	0.33	1.32	1.83	3.03	5.21	Null	Null	Null	Null	Null	Null	Null	–	–
30	Propanoic acid, 2-methyl-2,2-dimethyl-1-(2-hydroxy-1-methylethyl)propyl ester	0.25	1.45	1.62	2.21	3.73	Null	Null	Null	Null	Null	Null	Null	–	–
31	Propanoic acid, 2-methyl-1-(1,1-dimethylethyl)-2-methyl-1,3-propanediyl ester	0.28	0.69	1.30	2.95	4.07	Null	Null	Null	Null	Null	Null	Null	–	–
34	2,6-Bis(1,1-dimethylethyl)-4-(1-methylpropyl)-phenol	0.44	0.82	1.01	3.08	5.10	Null	Null	Null	Null	Null	Null	Null	Suspected insecticides	–
36	4-Octadecyl-morpholine	–	–	–	1.37	0.51	Null	Null	Null	Null	Null	Null	Null	Suspected pesticides	–

^a^Here the number was the same as Supplementary Annex S3, but the sorting was in descending order according to the aroma components' OAV of sample E.

^b^The relative content of each aroma component was calculated by peak area ratio (except *cis*-3-hexen-1-ol),'–' means undetected, and A represented sample A (spreading), B represented sample B (fixing), C, sample C (carding), D, sample D (pressing flat), E, sample E (drying).

^c^The OT of all aroma components came from Leffingwell and Associates data base (https://www.leffingwell.com/), and Compilations of Odour Threshold Values in Air, Water and other Media AND Compilations of Flavour Threshold Values in Water and other Media (2011 Editions)—by L.J. van Gemert (https://www.thresholdcompilation.com/), except for (Z)-3-methyl-2-(2-pentenyl)-2-cyclopenten-1-one, it was determined by our group, Null means not found or odorless.

^d^Reference means these discriptor was retrieved from FEMA GRAS data base (https://www.femaflavor.org/), except for linalool oxide, sniffing test, means the data came from the sniffing test.^e^COG, contribution of green aroma, which was determined by aroma monomer sniffing test, contributions were 1 and no contribution was 0, '–' means no testing.

^f1,f2^Data sources were Wang et al.^34^.

^g^In the table, the aroma components distinguished by the black line were group 1 (OAV > 1 in sample E), group 2 (OAV < 1 in sample E), group 3 (non aroma components), respectively.

Table 1 showed that 38 VOCs were detected and qualitatively identified in tea samples at different processing stages, and the relative content of other compounds could be calculated according to the peak area ratio because the content of *cis*-3-hexen-1-o in each tea sample was known.

OAV evaluation results (Table 1) showed that among the 38 VOCs identified by GC–MS, 32 were aroma components. For sample E, there were 25 aroma components of OAV > 1 (group 1), of which the largest was (Z)-3-methyl-2-(2-pentenyl)-2-Cyclopenten-1-one (5,000.00), the smallest was 1-Penten-3-ol (1.02), 7 aroma components of OAV < 1 (group 2), 6 odorless or non-aroma components (group 3). Among the 32 aroma components, 12 aromas contributed to green aroma were identified by the sensory evaluation group, according to the descending order of the OAV in sample E, they were geraniol (OAV = 192.00), hexanal (OAV = 116.00), (Z)-2-hexenal (59.00), (E)-2-hexenal (57.83), (E)-3-hexen-1-ol (39.14), citronellol (25.67), 1-hexanol (3.63), 1-penten-3-ol (1.02), *cis*-3-hexen-1-ol (0.20), 2-ethyl-1-hexanol (0.01), (Z)-2-penten-1-ol (0.00) and (E)-2-hexen-1-ol (0.00), respectively.

In addition, compared with sample A and sample E, among the 32 aroma components, the content of 16 aroma components increased, which were (Z)-3-methyl-2-(2-pentenyl)-2-cyclopenten-1-one, 1-octen-3-ol, 1-pentanol, (E)-2-heptenal, hexanal, 3,7-dimethyl-(Z)-2,6-octadienal, (E)-2-hexenal, methyl salicylate, β-myrcene, (E)-3-hexen-1-ol, benzaldehyde, (Z)-3,7-dimethyl-2,6-octadien-1-ol, 1-penten-3-ol, phenylethyl alcohol, furfural and 2-ethyl-1-hexanol, respectively, and the number of aroma components containing fruit aroma was the largest (9); and there were 9 aroma components with decreasing content, which were 3,7-dimethyl-1,6-octadien-3-ol, linalool oxide (pyranoid) (III and IIII), geraniol, (Z)-2-hexenal, 1-hexanol, *cis*-3-hexen-1-ol, 3-methyl-1-butanol, (Z)-2-penten-1-ol and (E)-2-hexen-1-ol respectively, and among them, the majority contained green (6); and there were 7 new aroma components generated in course of processing, which were Dodecanal, Linalool oxide (furanoid) (I and II), benzeneacetaldehyde, 1-ethyl-1H-pyrrole-2-carboxaldehyde, citronellol, tetradecanal, 1-hexadecanol, respectively, including 4 floral components and 3 sweet components.

In summary, the changes of aroma components and their contents in green tea samples during processing have been identified. However, E-nose test and flavor profile test were still needed to determine the changes of the overall aroma profile of green tea samples, and conducting association analysis with both of them, then whether the aroma profile of green tea samples is consistent with the changes of aroma components could be judged, as well as whether the most important aroma component affecting green aroma is *cis*-3-hexen-1-ol or not.

### Results of aroma profile, E-nose experiments and chemometrics analysis

The results of aroma profile experiment were shown in Fig. 2a. In terms of aroma characteristics of tea samples, the green (score = 2.75) and fruit (1.50) of spreading tea (sample A) were the strongest, the roasted (2.25) of fixing tea (sample B) was the strongest, and the sweet (2.25) and flower (2.08) of drying tea (sample E) were the strongest, this indicated that the aroma characteristics of tea samples changed dramatically during the processing, and the green odor is converted into green aroma between sample D and sample E, at this time, the content of *cis*-3-hexen-1-ol decreased from 0.52 to 0.04 mg kg^−1^, which indicated that when *cis*-3-hexen-1-ol was near this content, the green odor disappeared and the green aroma appeared.

In terms of the dynamic change of aroma profile, green aroma gradually decreased (2.75 → 0.67) in the whole processing process, and the score was less than 1 at the time of pressing flat (sample D, 0.92) and later, floral (0.92 → 2.08) and fruity (0.75 → 1.17) aroma increased gradually from the beginning of fixing, and herbal aroma fluctuated the least during the processing (0.33 → 0.58).

In addition, it should be noted that the difference of aroma profile between sample A and sample E was consistent with the difference of aroma activity components, that is the increase of the content of aroma components that contribute to the floral (4 aroma components), fruity (9 aroma components) and sweet (3 aroma components) aroma, and the decrease of the content of aroma components that contribute to the green (6 aroma components), and it had been analyzed in detail in “Detection results of GC–MS and calculation results of OAV”.

To summarize, the aroma characteristics of tea samples changed dramatically during the processing, and the main change was that the aroma of green and roasted declined, while that of floral, fruity, fresh, herbal and sweet rose. Moreover, the inflexion point of green odor transforming green aroma occurred after pressing flat. However, there were still some deficiencies in the qualitative analysis of tea aroma changes based on the aroma profile test, it is necessary to use E-nose to assist the test and try to characterize the change of its overall characteristics from another perspective.

The results of the E-nose test were shown in Fig. 2b. Compared with carding tea (sample C, sensing value_average_ = 0.2714) and drying tea (sample E, sensing value_average_ = 0.2570), pressing flat tea (sample D, sensing value_average_ = 0.1598) had a sharp decline in the sensing value of E-nose sensor, which was the lowest among all samples, and this was consistent with the results of aroma profile test, that is, the aroma components have changed significantly in pressing, the green odor disappeared and the green aroma appeared. In addition, the change rule of tea aroma in processing was evaluated by E-nose's radar map, which showed that it was irregular and repeated fluctuation, specifically, the 12 sensors of T series and P series all showed sample C > sample E > sample B > sample A > sample D, which those dramatic and repeated changes were consistent with the results of aroma profile test.

In addition, it can be seen from the overall E-nose radar profile of sample C and sample E that they had a high similarity, but in the aforementioned aroma profile test, they were quite different, this is because the sensing value of E-nose was an electrical signal generated based on the content of the chemical group (or ion) corresponding to the VOCs adsorbed by the sensor array (relative to the calibration reagent), it could be seen from Supplementary Annex S2 that E-nose sensors used in this test were all broad-spectrum sensors, they cannot specifically identify key aroma components such as *cis*-3-hexen-1-ol, but only chemical groups (or ions) such as fluoride (LY2/LG, P40/1, T40/1), chlorine (T30/1, P10/1, P40/1, P40/2, T40/2), therefore, it could only characterize the difference of chemical groups (or ions) of VOCs in different samples, so as to determine the difference of overall aroma, but not the source of the difference or quantitative analysis.

In fact, based on the above-mentioned E-nose detection principle, some common characteristics of tea could also be found. For example, the sensing values of all tea samples in the sensing area of LY2 (LY2/LG, LY2/G, LY2/AA, LY2/Gh, LY2/gCTl, LY2/gCT) were lower (− 0.0244 to 0.0616), this was related to the detection of air pollutants such as fluoride, chloride, oxynitride, sulfide, ammonia, hydrogen sulfide by the above sensors, and almost no such substances exist in natural tea samples, the similar cases also included the relatively low sensing values of tea samples in T40/2 (chloride) and T70/2 (toluene, xylene, carbon monoxide), obviously, there were very few corresponding substances in natural tea samples. Interestingly, the above two results were consistent with the E-nose detection of oolong tea by Zuobing Xiao et al.^17^, which also proved that it may be a universal attribute of tea.

Overall, through E-nose detection, it could be seen that the aroma of green tea changed dramatically in the whole processing, and the change was the largest before and after pressing flat (sample D), which was consistent with the results of aroma profile test, but it cannot be confirmed that these changes were caused by *cis*-3-hexen-1-ol or green aroma. Therefore, further chemometrics analysis was needed for the quantitative evaluation of aroma components.

The chemometrics analysis was divided into two steps. First, the correlation analysis was carried out to evaluate the correlation between OAV of 32 aroma activity components (group 1 and group 2 in Table 1) detected in tea samples and the green score in the aroma profile (Pearson correlation coefficient, two-tailed test), the results were shown in Table 2. It could be seen that OAV of 7 aroma components is significantly (or extremely significantly) related to the green score, among which 5 components were significantly positively related, namely 3,7-dimethyl-1,6-octadien-3-ol, geraniol, (z)-3-hexen-1-ol, 3-methyl-1-butanol and (E)-2-hexen-1-ol, and among the 5 components mentioned above, geraniol, (z)-3-hexen-1-ol and (E)-2-hexen-1-ol had been confirmed by the evaluation team to contribute to green aroma in the aroma description test (Table 1), therefore these 3 components could be considered as the direct contribution components of green aroma in green tea; then, the other 2 significantly related components were 3,7-dimethyl-1,6-octadien-3-ol (floral) and 3-methyl-1-butanol (burn, cocoa, malt), which suggested that they might have synergistic effect on green tea aroma; in addition, dodecanal (fat, oil, gardenia) and (E)-2-heptenal (almond, fat, fruit), which had significant negative correlation, suggested that they may had inhibitory effect on green tea aroma, but this needs further experimental verification.

**Table 2:** Correlation analysis results of OAV of each aroma contribution components and green score of aroma profile^a^.

Aroma contribution components	Pearson correlation^b^	Significance	Aroma contribution components	Pearson correlation	Significance
(Z)-3-Methyl-2-(2-pentenyl)-2-Cyclopenten-1-one	− 0.501	0.390	(E)-3-Hexen-1-ol	− 0.796	0.107
3,7-dimethyl-1,6-Octadien-3-ol	0.924*	**0.025**	1-Ethyl-1H-pyrrole-2-carboxaldehyde	− 0.507	0.383
Dodecanal	− 0.963**	**0.008**	Citronellol	− 0.654	0.232
Linalool oxide (pyranoid) (III and IV)	0.379	0.529	Benzaldehyde	− 0.688	0.199
1-Octen-3-ol	− 0.639	0.246	Tetradecanal	− 0.605	0.280
1-Pentanol	− 0.642	0.243	1-Hexanol	0.789	0.112
Linalool oxide (furanoid) (I and II)	− 0.704	0.184	(Z)-3,7-dimethyl-2,6-octadien-1-ol	− 0.495	0.397
Geraniol	0.887*	**0.045**	1-Hexadecanol	− 0.600	0.285
(E)-2-Heptenal	− 0.976**	**0.004**	1-Penten-3-ol	− 0.738	0.154
Hexanal	0.076	0.904	Phenylethyl alcohol	− 0.277	0.652
3,7-Dimethyl-(Z)-2,6-octadienal	− 0.268	0.663	Furfural	− 0.800	0.104
(Z)-2-Hexenal	0.321	0.598	***cis-3-Hexen-1-ol***	0.988**	**0.002**
(E)-2-Hexenal	− 0.104	0.868	2-Ethyl-1-hexanol	− 0.661	0.225
Benzeneacetaldehyde	− 0.507	0.383	**3-Methyl-1-Butanol**	0.940*	**0.018**
Methyl salicylate	− 0.283	0.645	(Z)-2-Penten-1-ol	0.142	0.820
β-Myrcene	0.049	0.938	**(E)-2-Hexen-1-ol**	0.930*	**0.022**

^a^The independent variable was the change of OAV of 32 aroma components from sample A to sample E, and the corresponding variable was the aroma profile score of green aroma.

^b^The correlation coefficient was Pearson coefficient; “*” means P < 0.05, “**” means P < 0.01.

Finally, stepwise linear regression analysis was carried out. OAV of 12 aroma components considered to have green aroma contribution in 5 tea samples (sample A to sample E) was taken as independent variable, and green score was taken as corresponding variable to carry out stepwise linear regression equation fitting, since the above 12 aroma components were assumed to be all possible sources of green aroma in tea samples, no constant was set during fitting, and the fitting equation was S_(green)_ = 0.073 × X_1_ + 0.014 × X_2_ (X_1_ = OAV (*cis*-3-hexen-1-ol), X_2_ = OAV ((E)-3-hexen-1-ol), R^2^ = 0.995, P = 0.000 < 0.05), this showed that the equation had metrological significance; in addition, two independent variables in the equation can pass the significance test (P < 0.05) and the variance inflation factor (VIF) test (VIF < 10), which showed that the equation fits well and has no multicollinearity, and the results showed that the OAV changes of *cis*-3-hexen-1-ol and (E)-3-hexen-1-ol were linearly correlated with the change of green aroma score, among which the coefficient of OAV (*cis*-3-hexen-1-ol) was higher (0.073), therefore *cis*-3-hexen-1-ol was the most influential component among the 12 aroma components contributing for green aroma.

In summary, 12 aroma components that contribute to green tea aroma were identified by sniffing of aroma monomer, among which 3 components were positively correlated with green aroma score through correlation analysis, and they were the direct contribution components of green aroma of green tea, finally, through stepwise linear regression analysis, the fitting equation of green aroma score was obtained, which was S_(green)_ = 0.073 × X_1_ + 0.014 × X_2_ (X_1_ = OAV (*cis*-3-hexen-1-ol), X_2_ = OAV ((E)-3-hexen-1-ol)), among which the coefficient of OAV (*cis*-3-hexen-1-ol) was higher (0.073), thus it was proved that *cis*-3-hexen-1-ol is the most direct contribution to the aroma of green tea.

### Effect of *cis*-3-hexen-1-ol on the aroma intensity of other aroma components in aroma monomer system

**Figure 3:**
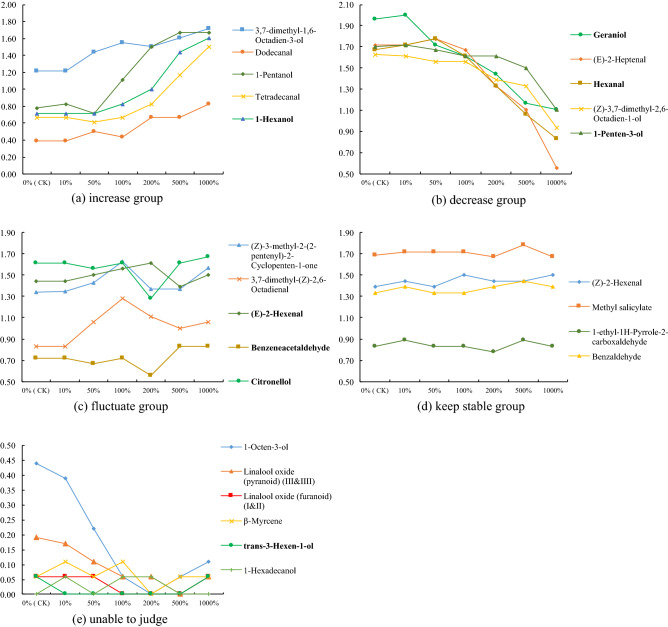
Effect of (Z)-3-hexen-1-ol on the aroma intensity of other aroma components in aroma monomer system. (**a**) The concentration of each aroma component was set to the concentration detected in sample E (Table 1); (**b**) the content of (Z)-3-hexen-1-ol in sample E was 100% (0.04 mg kg^−1^), thus "10%" was 0.004 mg kg^−1^, "50%" was 0.02 mg kg^−1^, "100%" was 0.04 mg kg^−1^, "200%" was 0.08 mg kg^−1^, "500%" was 0.2 mg kg^−1^, "1,000%" was 0.4 mg kg^−1^; (**c**) "Increase" means that the score of aroma intensity was significantly higher than CK at "1,000%", "decrease" means that the score was significantly lower than CK at "1,000%", "fluctuate" means that the score changed significantly during the process, but the difference between "1,000%" and CK was not significant, "keep stable" means that the difference of all concentrations is not significant; “unable to judge” means the score of the aroma component at all concentrations was less than 0.5, so the evaluation team can't accurately judge the change trend.

In order to find out how *cis*-3-hexen-1-ol affected the overall aroma of tea, it was necessary to studied the influence of *cis*-3-hexen-1-ol on 25 aroma activity components in the aroma monomer system, therefore, based on the aroma components and content in sample E, mixed the different concentrations of *cis*-3-hexen-1-ol with each aroma component in pairs, and sniffed the changes of aroma intensity. Saw Fig. 3 for the statistical results.

It could be seen that the effect of *cis*-3-hexen-1-ol on 25 aroma activity components could be divided into 4 groups, group I (increase), which included 5 aroma activity components, namely 3,7-dimethyl-1,6-octadien-3-ol, dodecanal, 1-pentanol, tetradecanal and 1-hexanol; group II (decrease), including 5 aroma active components, namely geraniol, (E)-2-heptenal, hexanal, (z)-3,7-dimethyl-2,6-octadien-1-ol and 1-penten-3-ol; group III(fluctuat), including 5 aroma activity components, namely (z)-3-methyl-2-(2-pentenyl)-2-cyclopenten-1-one, 3,7-dimethyl-(z)-2,6-octadienal, (E)-2-hexenal, benzeneacetaldehyde and citronellol; group IV (keep stable), including 4 aroma activity components, namely (z)-2-hexenal, methyl salicylate, 1-ethyl-1 h-pyrrole-2-carboxaldehyde and benzaldehyde. This showed that *cis*-3-hexen-1-ol could enhance group I aroma, inhibit group II aroma and interfere with group III aroma to achieve the effect on green tea aroma, however, the aroma of group IV was not affected by *cis*-3-hexen-1-ol.

In addition, it should be noted that the score of 6 aroma components was always less than 0.5, which means that they probably were odourless at the concentration of sample E, so it was impossible to judge the effect of *cis*-3-hexen-1-ol on them, however, this was not consistent with the results based on OAV evaluation, which may be related to the volatility of aroma threshold, that is, the thresholds of the same aroma (using the same solvent) determined by different sniffing groups may be different, so further correction may be needed to determine whether they were affected. At the same time, 4 of the 5 aroma components enhanced by *cis*-3-hexen-1-ol contributed to the floral or fruity aroma (3,7-dimethyl-1,6-octadien-3-ol, 1-pentanol, tetradecanal, 1-hexanol), which suggested that green aroma may have synergistic effect with the expression of floral and fruity aroma characteristics, and it could also explain the reason sample A had a high score of floral (1.83) and fruity score (1.50) (Fig. 2a). In addition, *cis*-3-hexen-1-ol did not have the general enhancement effect on other green aroma components as expected, in fact, among the 8 components that contributed to green, there were 3 decreased, which were geraniol, hexanal and 1-penten-3-ol respectively, 3 fluctuated, which were (E)-2-hexenal, benzeneacetaldehyde and citronellol respectively, and 1 couldn't judge which was *trans*-3-hexen-1-ol, only 1 increased which was 1-hexanol, it is speculated that the reason was that *cis*-3-hexen-1-ol has obvious grass and sharp characteristics, so it is easy to cause other aroma components to change or be inhibited, another similar component was methylis salicylas, which also had similar change or inhibitory effect on other aroma, however, this needs further experimental verification.

### Effect of *cis*-3-hexen-1-ol on the overall aroma of green tea in mixed systems

The influence of *cis*-3-hexen-1-ol on the aroma components has been studied in the monomer system test mentioned above. However, in the "natural mixed system" of tea, the influence of *cis*-3-hexen-1-ol may be different, so it needed to be verified in tea and the corresponding aroma reconstitution solution.

**Figure 4:**
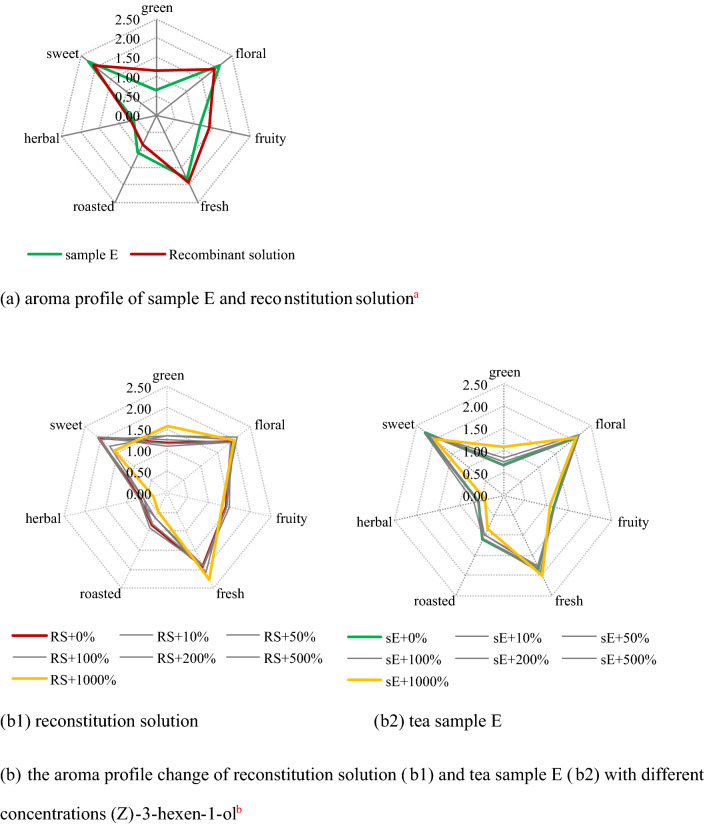
Aroma profile evaluation results of aroma reconstitution test and of exogenous (Z)-3-hexen-1-ol addition test in finished green tea. (**a**) Reconstitution solution were reconstituted according to the composition and content of 25 aroma components (Table 1, sample E, group 1) detected from sample E, and the substrate was distilled water; (**b**) “RS” means reconstitution solution, “sE” means sample E, + 0% to + 1,000% means the concentration of exogenous (Z)-3-hexen-1-ol in sniffing samples, of which + 0% was 0.00 mg kg^−1^, + 1,000% was 0.4 mg kg^−1^.

First, according to the composition and content of 25 aroma components (Table 2, sample E, group1) detected from sample E, the reconstituted aroma was reconstituted with redistilled water as a solvent (results were shown in Fig. 4a). It could be seen that compared with the sample E, the green (1.17) and fruity (1.42) aroma of the reconstitution solution were higher, while the sweet (2.08) and roasted (0.83) aroma were insufficient, this showed that although the overall aroma characteristics of sample E could be reappear to a certain extent according to the evaluation of aroma profile score, there were still significant differences between both of them, and the possible reasons will be further elaborated in the discussion.

Secondly, *cis*-3-hexen-1-ol with concentration of 10%, 50%, 100%, 200%, 500% and 1,000% (based on *cis*-3-hexen-1-ol content in sample E) was added into sample E and reconstituted solution respectively, and then the aroma profiles were evaluated (Fig. 4b).

The results showed that in terms of the overall change trend of aroma, the reconstitution solution and sample E showed the same trend, with the increased of *cis*-3-hexen-1-ol concentration, green (grass) (S_RS_ = 1.17 → 1.58, S_sE_ = 0.67 → 1.08) and fresh (S_RS_ = 1.92 → 2.25, S_sE_ = 1.83 → 2.00) were increasing, herbal (S_RS_ = 0.67 → 0.33, S_sE_ = 0.58 → 0.42), roasted (S_RS_ = 0.83 → 0.50, S_sE_ = 1.08 → 0.83) and sweet (S_RS_ = 2.08 → 1.58, S_sE_ = 2.25 → 2.00) were decreased continuously, but floral (S_RS_ = 1.92 → 2.00, S_sE_ = 2.08 → 2.08) and fruity (S_RS_ = 1.42 → 1.33, S_sE_ = 1.17 → 1.08) showed varying degrees of fluctuation, this showed that *cis*-3-hexen-1-ol could enhance green and fresh raoma, inhibit herbal, roasted and sweet, and interfere with floral and fruity to realize the effect on the overall aroma of green tea. In terms of the overall change range of aroma score, the total change range of aroma profile score of the reconstitution solution (29.56%) was larger than that of sample E (23.58%), which indicated that the influence of different concentrations of *cis*-3-hexen-1-ol on the reconstitution solution was greater than that of sample E, suggesting that there may be some substances in tea samples that can inhibit the aroma of *cis*-3-hexen-1-ol, or keep the original aroma characteristics, so that the aroma characteristics of sample E were more difficult to affect. In addition, the reconstitution solution showed green odor at 200% exogenous *cis*-3-hexen-1-ol (0.08 mg L^−1^), while sample E appeared at 1,000% (0.44 mg kg^−1^, 0.40 mg kg^−1^ exogenous + 0.04 mg kg^−1^ endogenous), which was consistent with the result of the disappearance of green odor between sample D (concentration of *cis*-3-hexen-1-ol was 0.52 mg kg^−1^) and sample E (concentration was 0.04 mg kg^−1^), it’s further proved that the threshold range of *cis*-3-hexen-1-ol was 0.04–0.52 mg kg^−1^ when the green odor changes into green aroma in tea, but the difference between the reconstitution solution and sample E also showed that the performance of green odor was not only determined by the concentration of (exogenous) *cis*-3-hexen-1-ol in tea samples, but also may influenced by some unknown "inhibitors" of green odor. In addition, regarding the difference between green aroma and green odor, the evaluation team believed that it was related to the "grass smelling" of *cis*-3-hexen-1-ol, which is consistent with previous research results, McRae Jeremy F's research^20^ suggested that the grass smelling of *cis*-3-hexen-1-ol may caused different odor receptor reactions with green aroma, which may leaded to unpleasant green odor.

Finally, tried to match the test results of the "natural mixed system" (Fig. 4b2) with the test results of the aroma monomer system (Fig. 4). The increase of green and fresh aroma may be related to the increase of the content of *cis*-3-hexen-1-ol and the synergistic enhancement of 1-hexanol (green, flower, unripe banana), while the decrease of sweet and the fluctuation of floral and fruity aroma may be related to the masked of geraniol (sweet, fruit, flower, lemon, green) and (z)-3,7-dimethyl-2,6-octadien-1-ol (sweet, fruit, flower, Lemon), and the weakening of herbal aroma may be affected by (z)-3-methyl-2-(2-pentenyl)-2-cyclopenten-1-one, however, the change of roasted aroma seemed unable to be explained by the aroma components in Fig. 4, which needed further experiment.

In summary, the effects of *cis*-3-hexen-1-ol on other aroma components and on the overall aroma of green tea were explored through aroma monomer system test and mixed system test, the results of the former test showed that there were 4 main modes of *cis*-3-hexen-1-ol on other aroma components, namely increase, decrease, fluctuate and keep stable; the results of the latter experiment showed that *cis*-3-hexen-1-ol mainly enhanced green and fresh, masked herbal and roasted, interfered and even masked sweet, floral and fruity to achieve the effect on the aroma of green tea, in addition, the performance of other aroma is consistent with that of aroma monomer test except roasted.

## Discussion

### Molecular structure basis of green aroma components in tea

12 aroma components which contributed to green aroma were identified from the natural aroma components of tea, and compared with their molecular structure, it could be found that there was a certain consistency. Therefore, the structural basis of the green aroma components of tea was discussed.

According to Amoore's “Stereochemical Theory of Odor”^21^, the aroma characteristics are affected by the functional groups and Stereochemical structure of the components. On this basis, it could be found that most of the structural units of the 12 components detected in this experiment had unsaturated carbon–carbon double bonds, and there were saturated or unsaturated oxygen atoms 1 or 3, and the carbon chains are all 8 carbon (or less) short chains. In addition, carbon–carbon double bond, hydroxyl group, carboxyl group and other functional groups were often considered as aroma generating groups in the study of aroma^22^, and when there were two functional groups with acceptable hydroxyl groups in the molecular structure in a relatively close position^23,24^, the aroma intensity was stronger.

Based on the Inelastic Electron Tunneling Spectroscopy (IETs) technology^25^, Turin proposed a new theory of aroma vibration^26^, it believe that the vibration energy of aroma components could be recognized by the olfactory signal receiver of human body, after that, he used IETs to analyze the components considered to have bitter almond aroma^27,28^, and found that hydrogen cyanide and benzaldehyde had characteristic peaks around 920 cm^−1^ and 2,000 cm^−1^, respectively. In terms of green aroma components, such as (E)-3-hexen-1-ol and (E)-2-hexen-1-ol, they were reported to have characteristic peaks only at 2,000 cm^−1^, but not at 920 cm^−1^. Therefore, it is possible that the IETs characteristic peak of green aroma is 2,000 cm^−1^, but it needs to be further verified by experiments.

In summarize, based on the Stereochemical Theory of Odor, it is speculated that the components of green aroma in tea are short carbon chain structure of 8 carbon (or less), and there are unsaturated carbon–carbon double bond and 1,3-position saturated (or unsaturated) oxygen atom; based on the new aroma vibration theory, it is speculated that the components of green aroma in tea are the components with characteristic peak at 2,000 cm^−1^ in IETs scanning.

### The difference between the results of reconstitution solution and tea sample test

In this experiment, there were some differences in aroma profile between the reconstitution solution and the tea sample. We speculate that this may be related to the reconstituted substrate composition and the potential contributing components which OAV < 1.

In the existing research, the aroma reconstitution of tea was often carried out with pure water as the substrate^29^, which is because the tea drinking way is to brew with boiling water. However, the results of this experiment and the reported recombination experiments showed that there were some differences between the reconstitution solution and the tea samples, which may came from not only the qualitative and quantitative detection errors, but also the interference of non-volatile substances in tea. Previous studies have shown that the aromatic strength of linalool (3,7-dimethyl-1,6-octadien-3-ol) may be inhibited by the esters in the substrate^30^, while the esters in tea may exceed 8% of the dry weight, which may lead to the inhibition of the aroma of 3,7-dimethyl-1,6-octadien-3-ol, so that the flower aroma score in the reconstitution solution was lower than that of the tea sample. And some soluble sugars may enhance the perception of sweet aroma, Isogai^31^ found that sucrose could significantly enhance the perception of sweet aroma, while the sucrose content in tea can generally reach 10^0^–10^1^ mg kg^−1^, which may lead to the weakening of sweet aroma in the reconstitution solution.

In addition, the selection of aroma components in the reconstitution solution was based on OAV, however, olfactory stimulation depends on the content and proportion of aroma components in the inhaled air, and the latter is affected by the volatilization rate and saturation of aroma substances from the substrate, which depends on the overall Saturated Vapor Pressure of the solution. Previous studies have shown that the composition and content of aroma components in aqueous solution will affect the saturated vapor pressure of the solution, and according to Raoult's law^32^, the higher the content of solute is, the higher the saturated vapor pressure is, the more volatile the solution is. However, the measurement of odor threshold of aroma monomer is carried out under the condition of single solute, and its saturated vapor pressure is not the same as that of the mixed solution. Therefore, the odor threshold measured by aroma monomer may be different from the mixed state of tea samples, and the odorless volatile components and nonvolatile components^33^ may also affect the volatile ability of aroma components.

Overall, the difference of aroma profile between the reconstitution solution and the tea sample may be due to the interference of non-volatile substances and odourless volatile substances in the tea sample, thus the reconstitution of tea aroma should consider the preparation of more complex substrate with higher reduction degree and the selection of some volatile components with OAV < 1.

## Conclusion

There were 32 VOCs that contribute to the aroma of green tea, 12 of which contributed to the aroma characteristics of green, they were geraniol, hexanal, (Z)-2-hexenal, (E)-2-hexenal, (E)-3-hexen-1-ol, citronellol, 1-hexanol, 1-penten-3-ol, *cis*-3-hexen-1-ol, 2-ethyl-1-hexanol, (Z)-2-penten-1-ol and (E)-2-hexen-1-ol, respectively. Among the above 12 components, 3 were positively correlated with the green score of aroma profile, they were geraniol, *cis*-3-hexen-1-ol and (E)-2-hexen-1-ol, respectively. Further fitting stepwise linear regression equation, was S (green) = 0.073 × OAV (*cis*-3-hexen-1-ol) + 0.014 × OAV ((E)-3-hexen-1-ol) (R^2^ = 0.995, P = 0.000 < 0.01). Therefore, *cis*-3-hexen-1-ol played the leading role in green aroma of green tea.

There were four types of effect with *cis*-3-hexen-1-ol on other 19 aroma in green tea, the first was increase effect (5 aroma components), including 3,7-dimethyl-1,6-octadien-3-ol, dodecanal, 1-pentanol, tetradecanal and 1-hexanol; the second was decrease effect (5 aroma components), including geraniol, (E)-2-heptenal, hexanal, (Z)-3,7-dimethyl-2,6-octadien-1-ol and 1-penten-3-ol; the third was fluctuate effect (5 aroma components), including (Z)-3-methyl-2-(2-pentenyl)-2-cyclopenten-1-one, 3,7-dimethyl-(Z)-2,6-octadienal, (E)-2-hexenal, benzeneacetaldehyde and citronellol; the forth was keep stable effect (4 aroma components), including (Z)-2-hexenal, methyl salicylate, 1-ethyl-1H-pyrrole-2-carboxaldehyde and benzaldehyde. At the same time, the above changes could partly explain the effect of *cis*-3-hexen-1-ol on the overall aroma of green tea, that is to say, with the increase of exogenous *cis*-3-hexen-1-ol concentration, green and fresh of green tea increased, herbal, roasted and sweet decreased, floral and fruit fluctuated.

When green tea changed from green odor to green aroma, the concentration of endogenous *cis*-3-hexen-1-ol varied from 0.04 to 0.52 mg kg^−1^, in addition, the critical concentration of conversion between green odor and green aroma was 0.08 mg L^−1^ in the aroma reconstitution solution and 0.44 mg kg^−1^ (0.40 mg kg^−1^ exogenous + 0.04 mg kg^−1^ endogenous) in the tea samples.

## Supplementary information

Supplementary file1

## Data Availability

Correspondence and requests for materials should be addressed to X. D.

## Abbreviations

VOCs: Volatile organic compounds
OAV: Odor activity value
HS-SPME: Head-space solid-phase microextraction
GC–MS: Gas chromatography-mass spectrometry
GC-FID: Gas chromatography–flame ionization detector
E-nose: Electronic nose
OT: Odor threshold
