# Supplementary material for: Characterization of the effect of cis-3-hexen-1-ol on green tea aroma
Source: Sci Rep. 2020 Sep 23;10:15506. doi: 10.1038/s41598-020-72495-5 (PMC7511323; doi:10.1038/s41598-020-72495-5)
Supplement: Supplementary file 1 — Supplementary file1 [file 41598_2020_72495_MOESM1_ESM.docx]

**Characterization of the Effect of *cis*-3-hexen-1-ol on Green Tea Aroma**

Cong–ning Nie^1^, Yuan Gao^1^, Xiao Du^1^*, Jin-lin Bian^1^, Hui Li^2^, Xiang Zhang^1^, Cong-ming Wang^1^, Shun-yu Li^1^

^1^Sichuan Agricultural University, No. 211 Huimin Road, Wenjiang District, Chengdu, Sichuan, China

^2^Chengdu Agricultural College, No. 392 Detongqiao Road, Wenjiang District, Chengdu, Sichuan, China

***Corresponding authors: Prof. Dr. Xiao Du**

Sichuan Agricultural University, No. 211 Huimin Road, Wenjiang District, Chengdu, Sichuan 610000, China

E–mail: 10669@sicau.edu.cn


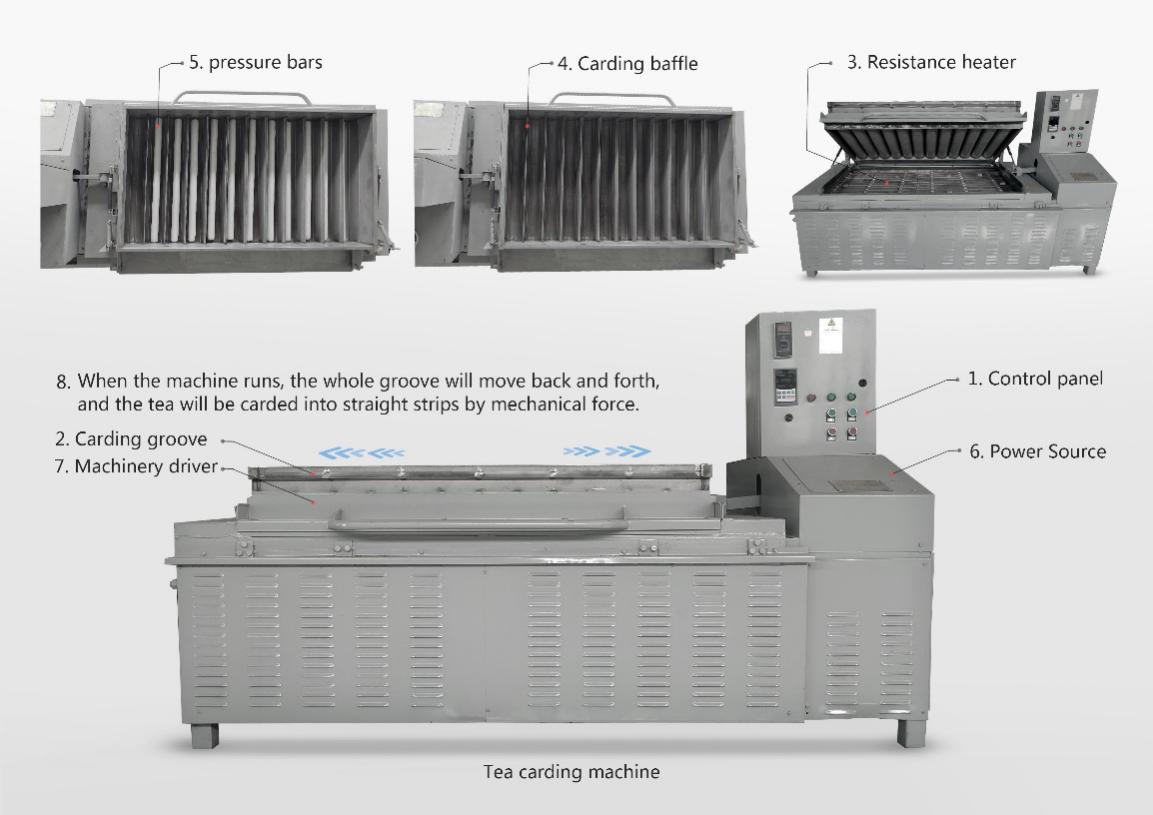


**Annex 1 Tea carding machine diagram**

Notes: 1. Control panel; 2. Carding groove; 3. Resistance heater; 4. Carding baffle; 5. Pressure bars; 6. Power source; 7. Machinery driver; 8. When the machine runs, the whole groove will move back and forth, and the tea will be carded into straight strips by mechanical force.

| **Annex 2 Detector information of the E-nose (Qin et al., 2013)^1^** | | |
| --- | --- | --- |
| Number | Sensor | Object substances for sensing |
| 1 | LY2/LG | Fluoride, chloride, oxynitride, sulfide |
| 2 | LY2/G | Ammonia, amines, carbon, oxygen compounds |
| 3 | LY2/AA | Alcohol, acetone, ammonia |
| 4 | LY2/Gh | Ammonia, amine compounds |
| 5 | LY2/gCTl | Hydrogen sulfide |
| 6 | LY2/gCT | Propane, butane |
| 7 | T30/1 | Polar compound, hydrogen chloride |
| 8 | P10/1 | Nonpolar compound: hydrocarbon, ammonia, chlorine |
| 9 | P10/2 | Nonpolar compound: methane, ethane |
| 10 | P40/1 | Fluorine, chlorine |
| 11 | T70/2 | Toluene, xylene, carbon monoxide |
| 12 | PA/2 | Ethanol, ammonia, amine compounds |
| 13 | P30/1 | Hydrocarbons, ammonia, ethanol |
| 14 | P40/2 | Chlorine, hydrogen sulfide, fluoride |
| 15 | P30/2 | Hydrogen sulfide, ketone |
| 16 | T40/2 | Chlorine |
| 17 | T40/1 | Fluorine |
| 18 | TA/2 | Ethanol |

| **Annex 3 Determination of aroma components in green tea samples by GC-MS** | | | | | | | | | | | | | | | |  |
| --- | --- | --- | --- | --- | --- | --- | --- | --- | --- | --- | --- | --- | --- | --- | --- | --- |
| No | aroma components | retention time (min) | RIs (DB-WAX) | | | Identification^c^ | peak area (%)^d^ | | | | | | | | | |
|  |  |  | calculate^a^ | reference^b^ | Literatures |  | sample A | | sample B | | sample C | | sample D | | sample E | |
|  |  |  |  |  |  |  | average | RSD | average | RSD | average | RSD | average | RSD | average | RSD |
| 1 | Hexanal | 3.938 | 1077 | 1078 | Lopes, Strobl, et al., 2004^2^ | MS, RI | 1.26 | 4.59 | 0.98 | 7.56 | 0.72 | 4.53 | 0.78 | 4.86 | 0.87 | 5.65 |
| 2 | β-Myrcene | 5.599 | 1164 | 1164 | Lopes, Strobl, et al., 2004^2^ | MS, RI | 1.40 | 6.42 | 0.28 | 5.32 | 1.36 | 2.85 | 1.01 | 5.87 | 1.00 | 8.43 |
| 3 | 1-Penten-3-ol | 6.388 | 1174 | 1174 | Gurbuz O., Rouseff J.M., et al., 2006^3^ | MS, RI | 0.38 | 10.40 | 0.25 | 10.60 | 3.54 | 2.57 | 5.39 | 5.63 | 4.58 | 9.08 |
| 4 | (E)-2-Hexenal | 6.926 | 1206 | 1205 | Osorio, Alarcon, et al., 2006^4^ | MS | 4.73 | 4.06 | 5.72 | 5.16 | 5.28 | 3.47 | 3.25 | 7.91 | 3.47 | 4.55 |
| 5 | (Z)-2-Hexenal | 7.416 | 1207 | 1207 | Canuti, Conversano, et al., 2009^5^ | MS, RI | 7.83 | 8.58 | 10.18 | 5.18 | 1.37 | 7.06 | 3.58 | 5.79 | 3.54 | 7.21 |
| 6 | 3-methyl-1-Butanol | 7.763 | 1217 | 1217 | Botelho, Caldeira, et al., 2007^6^ | MS, RI | 0.36 | 8.04 | 0.10 | 6.02 | － | － | － | － | － | － |
| 7 | 1-Pentanol | 9.403 | 1252 | 1252 | Cho, Namgung, et al., 2008^7^ | MS, RI | 0.24 | 8.40 | 9.56 | 7.02 | 9.14 | 6.00 | 7.17 | 5.72 | 1.98 | 3.68 |
| 8 | (E)-2-Heptenal | 11.058 | 1325 | 1323 | Hallier, Prost, et al., 2005^8^ | MS, RI | 0.50 | 5.58 | 3.73 | 10.51 | 3.21 | 7.45 | 3.77 | 6.38 | 2.88 | 8.62 |
| 9 | (Z)-2-Penten-1-ol | 11.613 | 1331 | 1332 | Rochat, Egger, et al., 2009^9^ | MS, RI | 0.43 | 10.94 | 2.13 | 8.75 | 1.49 | 7.67 | － | － | － | － |
| 10 | 1-Hexanol | 12.888 | 1360 | 1360 | Cho, Namgung, et al., 2008^7^ | MS, RI | 7.00 | 7.22 | 0.81 | 6.10 | 0.25 | 2.79 | 1.20 | 5.66 | 1.36 | 4.33 |
| 11 | (E)-3-Hexen-1-ol | 13.245 | 1371 | 1371 | Pozo-Bayon M.A., Ruiz-Rodriguez A., et al., 2007^10^ | MS, RI | 0.74 | 9.52 | 10.10 | 9.21 | 12.66 | 6.17 | 7.74 | 6.17 | 4.11 | 5.71 |
| 12 | *cis*-3-hexen-1-ol | 13.782 | 1381 | 1383 | Botelho, Caldeira, et al., 2007^6^ | MS, RI | 18.64 | 7.30 | 7.25 | 4.76 | 5.43 | 3.86 | 1.22 | 2.41 | 0.18 | 9.39 |
| 13 | (E)-2-Hexen-1-ol | 14.892 | 1394 | 1394 | Osorio, Alarcon, et al., 2006^4^ | MS, RI | 3.64 | 6.25 | 2.58 | 8.12 | 1.38 | 4.33 | － | － | － | － |
| 14 | Furfural | 16.64 | 1447 | 1443 | Guo, Wu, et al., 2008^11^ | MS | 0.40 | 4.49 | 1.27 | 5.85 | 3.09 | 5.03 | 3.06 | 1.91 | 3.19 | 3.91 |
| 15 | 1-Octen-3-ol | 16.761 | 1448 | 1448 | Guillot, Peytavi, et al., 2006^12^ | MS, RI | 0.49 | 10.37 | 1.44 | 5.84 | 2.70 | 2.73 | 2.84 | 6.71 | 0.87 | 5.75 |
| 16 | Linalool oxide (pyranoid) (III&IIII) | 17.104 | 1450 | Null | Null | MS | 5.46 | 10.83 | 5.79 | 5.88 | 8.98 | 4.60 | 2.16 | 7.58 | 2.82 | 2.96 |
| 17 | Linalool oxide (furanoid) (I&II) | 17.171 | 1451 | 1451 | Mahattanatawee K., Perez-Cacho P.R., et al., 2007^13^ | MS, RI | － | － | － | － | 1.48 | 7.62 | 1.36 | 6.13 | 2.03 | 5.66 |
| 18 | 2-Ethyl-1-hexanol | 18.325 | 1483 | 1483 | Karlsson, Birgersson, et al., 2009^14^ | MS, RI | 0.39 | 7.89 | 0.93 | 8.16 | 1.16 | 8.20 | 1.34 | 8.26 | 2.69 | 2.78 |
| 19 | Benzaldehyde | 18.584 | 1530 | 1530 | Cho, Namgung, et al., 2008^7^ | MS, RI | 0.32 | 8.51 | 0.78 | 6.20 | 2.52 | 3.21 | 3.94 | 2.35 | 6.38 | 4.73 |
| 20 | 3,7-dimethyl-1,6-Octadien-3-ol | 20.596 | 1548 | 1547 | Cho, Namgung, et al., 2008^7^ | MS, RI | 17.71 | 8.28 | 11.32 | 3.99 | 7.42 | 7.43 | 2.42 | 3.02 | 3.23 | 7.12 |
| 21 | 1-ethyl-1H-Pyrrole-2-carboxaldehyde | 22.011 | 1618 | 1616 | Welke, Manfroi, et al., 2012^15^ | MS, RI | － | － | － | － | － | － | － | － | 1.63 | 3.81 |
| 22 | Benzeneacetaldehyde | 23.307 | 1648 | 1648 | Cho, Namgung, et al., 2008^7^ | MS, RI | － | － | － | － | － | － | － | － | 0.75 | 1.33 |
| 23 | 3,7-dimethyl-(Z)-2,6-Octadienal | 24.845 | 1673 | 1670 | Cai, Lin, et al., 2006^16^ | MS, RI | 2.63 | 9.19 | 1.31 | 5.60 | 1.26 | 4.49 | 2.34 | 9.97 | 2.81 | 7.47 |
| 24 | Dodecanal | 26.346 | 1729 | 1729 | Gurbuz O., Rouseff J.M., et al., 2006^3^ | MS, RI | － | 5.25 | 1.38 | 8.90 | 1.75 | 6.70 | 1.26 | 5.73 | 1.09 | 6.41 |
| 25 | Methyl salicylate | 27.899 | 1747 | 1747 | Osorio, Alarcon, et al., 2006^3^ | MS, RI | 3.73 | 5.21 | 3.52 | 2.86 | 2.48 | 5.48 | 1.97 | 6.46 | 4.70 | 5.72 |
| 26 | Citronellol | 28.155 | 1764 | 1764 | Zhao, Xu, et al., 2009^17^ | MS, RI | － | － | 0.24 | 6.25 |  | 7.13 | 0.76 | 3.76 | 1.54 | 3.66 |
| 27 | (Z)-3,7-dimethyl-2,6-Octadien-1-ol | 29.697 | 1796 | 1797 | Zhao, Xu, et al., 2009^17^ | MS, RI | 0.46 | 5.79 | 1.26 | 4.01 | 0.05 | 9.50 | 0.48 | 2.75 | 0.93 | 9.51 |
| 28 | Geraniol | 31.437 | 1895 | 1895 | Zeng, Xie, et al., 2011^18^ | MS, RI | 14.96 | 7.15 | 2.89 | 3.74 | 2.41 | 8.42 | 2.25 | 5.00 | 2.16 | 1.64 |
| 29 | Propanoic acid, 2-methyl-3-hydroxy-2,4,4-trimethyl-pentyl ester | 31.855 | 1897 | Null | Null | MS | 0.85 | 8.59 | 4.26 | 8.59 | 5.87 | 3.44 | 7.11 | 8.65 | 7.81 | 2.26 |
| 30 | Propanoic acid, 2-methyl-2,2-dimethyl-1-(2-hydroxy-1-methylethyl)propyl ester | 32.037 | 1900 | Null | Null | MS | 0.65 | 4.10 | 4.68 | 5.80 | 5.22 | 5.56 | 5.18 | 3.37 | 5.60 | 3.50 |
| 31 | Propanoic acid, 2-methyl-1-(1,1-dimethylethyl)-2-methyl-1,3-propanediyl ester | 32.307 | 1903 | Null | Null | MS | 0.73 | 7.16 | 2.22 | 5.74 | 4.19 | 2.33 | 6.92 | 7.29 | 6.11 | 8.10 |
| 32 | Phenylethyl Alcohol | 32.949 | 1906 | 1905 | Karlsson, Birgersson, et al., 2009^14^ | MS, RI | 2.50 | 4.92 | － | － | － | － | － | － | 5.72 | 6.48 |
| 33 | (Z)-3-methyl-2-(2-pentenyl)-2-Cyclopenten-1-one | 33.714 | 1980 | 1984 | Wei A. and Shibamoto T., 2007^19^ | MS, RI | 0.43 | 8.14 | 0.41 | 2.67 | 0.34 | 5.41 | 0.44 | 3.33 | 2.32 | 3.18 |
| 34 | 2,6-bis(1,1-dimethylethyl)-4-(1-methylpropyl)-Phenol | 34.295 | 2045 | Null | Null | MS | 1.14 | 9.86 | 2.63 | 9.34 | 3.25 | 2.23 | 7.23 | 4.38 | 7.65 | 3.54 |
| 35 | Tetradecanal | 40.712 | 2181 | 1931 | Schirack, Drake, et al., 2006^20^ | MS | － | － | － | － | － | － | 2.27 | 4.10 | 0.80 | 5.25 |
| 36 | 4-octadecyl-Morpholine | 47.961 | 2337 | Null | Null | MS | － | － | － | － | － | － | 3.21 | 5.33 | 0.76 | 6.99 |
| 37 | 1-Hexadecanol | 48.095 | 2366 | 2363 | Osorio, Alarcon, et al., 2006^4^ | MS, RI | － | － | － | － | － | － | 0.60 | 7.72 | 1.72 | 5.96 |
| 38 | 1-Nonadecanol | 53.873 | 2698 | 2687 | Hanai and Hong, 1989^21^ | MS | － | － | － | － | － | － | 5.75 | 1.51 | 0.72 | 1.76 |
| a Calculate,Retention indices (RIs) were calculated against n-alkanes (C4–C20) (on DB-WAX column); | | | | | | | | | | | | | | | | |
| b Null, no relevant references or data were found; | | | | | | | | | | | | | | | | |
| c Identification, means method of identification. MS, mass spectrum comparison using NIST library; RI: retention index in agreement; | | | | | | | | | | | | | | | | |
| d RSD, relative standard deviation, which is standard deviation/arithmetical average; －,means not detected. | | | | | | | | | | | | | | | | |

**Reference**

1. Qin, Z. , Pang, X. , Chen, D. , Chen, H. , & Wu, J. . (2013). Evaluation of chinese tea by the electronic nose and gas chromatography-mass spectrometry: correlation with sensory properties and classification according to grade level. *Food Research International,* *52*(2), 864–874.

2. Lopes, D., Strobl, H., Kolodziejczyk, P., (2004). 14-Methylpentadecano-15-lactone (Muscolide): a new macrocyclic lactone from the oil of Angelica archangelica L., *Chemistry and Biodiversity*, 1, 12, 1880-1887

3. Gurbuz O., Rouseff J.M., Rouseff R.L.. (2006). Comparison of aroma volatiles in commercial Merlot and Cabernet Sauvignon wines using gas chromatography - Olfactometry and gas chromatography - Mass spectrometry, *J. Agric. Food Chem.*, 54, 11, 3990-3996

4. Osorio, C., Alarcon, M., Moreno, C., Bonilla, A., Barrios, J., Garzon, C., Duque, C.. (2006). Characterization of Odor-Active Volatiles in Champa ( Campomanesia lineatifolia R. P.), *J. Agric. Food Chem.*, 54, 2, 509-516

5. Canuti, V., Conversano, M., Li Calzi, M., Heymann, H., Matthews, M.A., Ebeler, S.E.. (2009). Headspace solid-phase microextraction - gas chromatography - mass spectrometry for profiling free volatile compounds in Cabernet Sauvignon grapes and vines, *J. Chromatogr. A.*, 1216, 15, 3012-3022

6. Botelho, G., Caldeira, I., Mendes-Faia, A., Clímaco, M.C.. (2007). Evaluation of two quantitative gas chromatography-olfactometry methods for clonal red wines differentiation, *Flavour Fragr. J.*, 22, 5, 414-420

7. Cho, I.H., Namgung, H.-J., Choi, H.-K., Kim, Y.-S.. (2008). Volatiles and key odorants in the pileus and stipe of pine-mushroom (Tricholoma matsutake Sing.), *Food Chem.*, 106, 1, 71-76

8. Hallier, A., Prost, C., Serot, T.. (2005). Influence in rearing conditions on the volatile compounds of cooked fillets of Silurus glanis (European catfish), *J. Agric. Food Chem.*, 53, 18, 7204-7211

9. Rochat, S., Egger, J., Chaintreau, A.. (2009). Strategy for the identification of key odorants: application to shrimp aroma, *J. Chromatogr. A*, 1216, 36, 6424-6432

10. Pozo-Bayon M.A., Ruiz-Rodriguez A., Pernin K., Cayot N.. (2007). Influence of eggs on the aroma composition of a sponge cake and on the aroma release in model studies on flavored sponge cakes, *J. Agric. Food Chem*., 55, 4, 1418-1426

11. Guo, L., Wu, J.-Z., Han, T., Cao, T., Rahman, K., Qin, L.-P.. (2008). Chemical composition, antifungal and antitumor properties of ether extracts of Scapania verrucosa Heeg. and its endophytic fungus Chaetomium fusiforme, *Molecules*, 13, 9, 2114-2125

12. Guillot, S., Peytavi, L., Bureau, S., Boulanger, R., Lepoutre, J.-P., Crouzet, J., Schorr-Galindo, S.. (2006). Aroma characterization of various apricot varieties using headspace-solid phase microextraction combined with gas chromatography-mass spectrometry and gas chromatography-olfactometry, *Food Chem.*, 96, 1, 147-155

13. Mahattanatawee K., Perez-Cacho P.R., Davenport T., Rouseff R.. ( 2007). Comparison of three lychee cultivar odor profiles using gas chromatography-olfactometry and gas chromatography-sulfur detection, *J. Agric. Food Chem.*, 55, 5, 1939-1944

14. Karlsson, M.F., Birgersson, G., Prado, A.M.C., Bosa, F., Bengtsson, M., Witzgall, P.. (2009). Plant Odor Analysis of Potato: Responce of Guatemalan Moth to Above- and Background Potato Volatiles, *J. Agric. Food Chem.*, 57, 13, 5903-5909

15. Welke, J.E., Manfroi, V., Zanus, M., Lazarotto, M., Zini, C.A.. (2012). Characterization of the volatile profile of Brazilian merlot wines through comprehensive two dimensional gas chromatography time-of-flight mass spectrometric detection, *J. Chromatogr. A*, 1226, 124-139

16. Cai, J., Lin, P., Zhu, X., Su, Q.. (2006). Comparative analysis of clary sage (S. sclarea L.) oil volatiles by GC-FTIR and GC-MS, *Food Chem.*, 99, 2, 401-407

17. Zhao, Y., Xu, Y., Li, J., Fan, W., Jiang, W.. (2009). Profile of volatile compounds in 11 brandies by headspace solid-phase microextraction followed by gas chromatography-mass spectrometry, *J. Food. Sci.*, 74, 2, c90-c99

18. Zeng, Z., Xie, R., Zhang, T., Zhang, H., Chen, J.Y.. (2011). Analysis of volatile compositions of Magnolia biondii Pamp by steam distillation and Headspace solid phase micro-extraction, *J. Oleo Sci.*, 60, 12, 591-596

19. Wei A., Shibamoto T.. (2007). Antioxidant activities and volatile constituents of various essential oils, *J. Agric. Food Chem.*, 55, 5, 1737-1742

20. Schirack, A.V., Drake, M.A., Sander, T.H., Sandeep, K.P.. (2006). Characterization of aroma-active compounds in microwave blanched peanuts, *J. Food Sci.*, 71, 9, c513-c520

21. Hanai, T., Hong, C.. (1989). Structure-retention correlation in CGC, *J. Hi. Res. Chromatogr.*, 12, 5, 327-332
